# Non-Invasive Imaging of Cysteine Cathepsin Activity in Solid Tumors Using a ^64^Cu-Labeled Activity-Based Probe

**DOI:** 10.1371/journal.pone.0028029

**Published:** 2011-11-21

**Authors:** Gang Ren, Galia Blum, Martijn Verdoes, Hongguang Liu, Salahuddin Syed, Laura E. Edgington, Olivier Gheysens, Zheng Miao, Han Jiang, Sanjiv Sam Gambhir, Matthew Bogyo, Zhen Cheng

**Affiliations:** 1 Molecular Imaging Program at Stanford (MIPS), Stanford University, Stanford, California, United States of America; 2 Department of Radiology, Stanford University, Stanford, California, United States of America; 3 Department of Bioengineering, Stanford University, Stanford, California, United States of America; 4 Department of Pathology, Stanford University, Stanford, California, United States of America; 5 Department of Microbiology and Immunology, Stanford University, Stanford, California, United States of America; 6 Bio-X Program, Stanford University, Stanford, California, United States of America; 7 Institute of Drug Research, The Hebrew University, Jerusalem, Israel; Genentech, United States of America

## Abstract

The papain family of cysteine cathepsins are actively involved in multiple stages of tumorigenesis. Because elevated cathepsin activity can be found in many types of human cancers, they are promising biomarkers that can be used to target radiological contrast agents for tumor detection. However, currently there are no radiological imaging agents available for these important molecular targets. We report here the development of positron emission tomography (PET) radionuclide-labeled probes that target the cysteine cathepsins by formation of an enzyme activity-dependent bond with the active site cysteine. These probes contain an acyloxymethyl ketone (AOMK) functional group that irreversibly labels the active site cysteine of papain family proteases attached to a 1,4,7,10-tetraazacyclododecane-1,4,7,10-tetraacetic acid (DOTA) tag for labeling with ^64^Cu for PET imaging studies. We performed biodistribution and microPET imaging studies in nude mice bearing subcutaneous tumors expressing various levels of cysteine cathepsin activity and found that the extent of probe uptake by tumors correlated with overall protease activity as measured by biochemical methods. Furthermore, probe signals could be reduced by pre-treatment with a general cathepsin inhibitor. We also found that inclusion of a Cy5 tag on the probe increased tumor uptake relative to probes lacking this fluorogenic dye. Overall, these results demonstrate that small molecule activity-based probes carrying radio-tracers can be used to image protease activity in living subjects.

## Introduction

Proteases play important roles in the regulation of both normal and disease processes. In particular, the papain family cysteine cathepsins are frequently over-expressed in a number of human cancers [1]-[7]. In addition, expression of a number of cysteine cathepsin including cathepsins B and L is increased in pre-neoplastic lesions and changes in both localization and sub-cellular distribution of these proteases are often observed in tumors [1], [3], [7]. This combination makes them potentially valuable cancer biomarkers [1]–[5], [8], [9]. Novel imaging methods and molecularly targeted tracers can now be used not only to locate a tumor, but also to visualize the expression and activity of specific molecular targets and biological processes in a tumor [10], [11]. These imaging methods have the potential to facilitate both early disease detection and to aid in the process of drug development by allowing non-invasive monitoring of target inhibition. Directed targeting of enzymatic proteins such as proteases using imaging agents also has the potential to provide greater detail about the basic biological framework of a tumor and provide better resolution of the disease phenotype [10], [12]–[16].

Since most proteases are initially synthesized as inactive zymogens that are activated by a complex set of post-translational mechanisms, tools that report on enzyme activity rather than protein abundance will be required to fully understand their function in complex biological processes. For this reason, a number of recent studies have focused on the development of imaging agents that act as substrates for a target protease [10], [13], [15], [16]. In addition, fluorescent molecules that only penetrate a cellular membrane when processed by a target protease have been developed [17]–[19]. While all of these methods have provided valuable new tools, none of these methods have been translated for use in radiological imaging.

We report here the development and application of radio-labeled small molecule activity-based probes (ABPs) that can be used for positron emission tomography (PET) imaging of cysteine cathepsin activity. These probes are based on the peptide acyloxymethyl ketones (AOMKs) that have been reported to be highly selective labels of a number of classes of cysteine cathepsins [20]. This class of reagents has also recently been used for optical imaging of cysteine cathepsin activity in live cells [8] and in near-infrared fluorescent (NIRF) labeled form for non-invasive optical imaging of cysteine cathepsin activity in living subjects [21], [22]. The probes presented here couple the intrinsic advantages of nuclear imaging, including high sensitivity, capability of quantification, and clinical translation, with the specific, covalent nature of these imaging agents. In addition, their relatively small size compared to substrate based probes provides favorable *in vivo* pharmacodynamic properties and cellular uptake. Thus, probes presented here represent potentially valuable new imaging tools for application to a number of clinically relevant diseases that involve increased cysteine cathepsin activity.

## Results

### Generation of a PET Probe Based On the GB123 Structure

**Figure 1:**
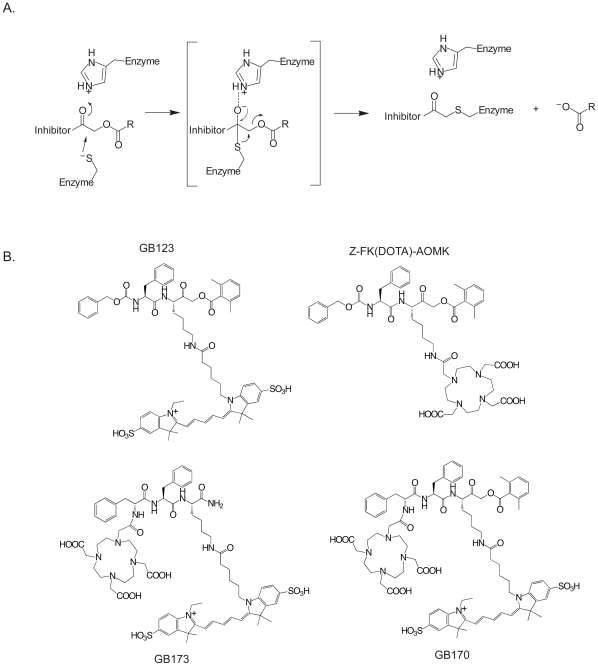
Mechanism and structures of activity-based imaging probes. (**A**) Mechanism of covalent inhibition of a cysteine protease by an acyloxymethyl ketone. (**B**) Structures of the activity based probes GB123, Z-FK(DOTA)-AOMK, GB170 and GB173.

ABPs provide an indirect readout of protease activity in complex proteomes. A number of ABPs targeting various cysteine cathepsins have recently been reported [20], [21], [23], [24]. In particular, the peptide acyloxymethyl ketones have been validated in optical imaging applications [8], [21], [22], [25]. The covalent modification of a cysteine protease by an AOMK results in alkylation of the active site thiol and loss of acyloxy group (Figure 1A). One of the initially reported AOMK derivatives, Z-FR-AOMK, exhibited high potency and specificity for cathepsin B and L [20]. Moreover, conversion of Z-FR-AOMK to the related Z-FK-AOMK and modification of the lysine side chain with bulky organic fluorescent dye resulted in a probe, GB123, that could be used to non-invasively image cathepsins B and L activity [8], [21]. Therefore, we synthesized a probe in which the fluorescent reporter of GB123 was replaced by the 1, 4, 7, 10-tetraazacyclododecane-1, 4, 7, 10-tetraacetic acid (DOTA) for coordination of the ^64^Cu tracer (Z-FK(DOTA)-AOMK; Figure 1B). We also synthesized a probe in which the original carboxylbenzoyl (Z) capping group was replaced by the DOTA group and an additional phenylalanine amino acid was added to mimic the properties of the Z group that was lost (GB170; Figure 1B). Finally, we generated a control analog that lacked the reactive AOMK electrophile (GB173; Figure 1B).

We radiolabeled the Z-FK(DOTA)-AOMK, GB-173 and GB-170 conjugates with ^64^Cu (t_1/2_ = 12.7 h, E_β_^+^_max_ = 656 keV, 19%) at 50 ^0^C for 1 h and purified the resulting probes to greater than 95% radiochemical purity using analytical RP-HPLC (Figure S1). The specific radioactivity of ^64^Cu-Z-FK(DOTA)-AOMK was 0.48 Ci/µmol (17.8 MBq/nmol, 500 µCi/µg) at the end of synthesis (EOS). The specific radioactivity of GB170 and GB173 were determined to be around 20 µCi/µg EOS. These labeled compounds were then used for *in vitro* and *in vivo* labeling experiments.

### Labeling properties of ^64^Cu labeled AOMK analogs

**Figure 2:**
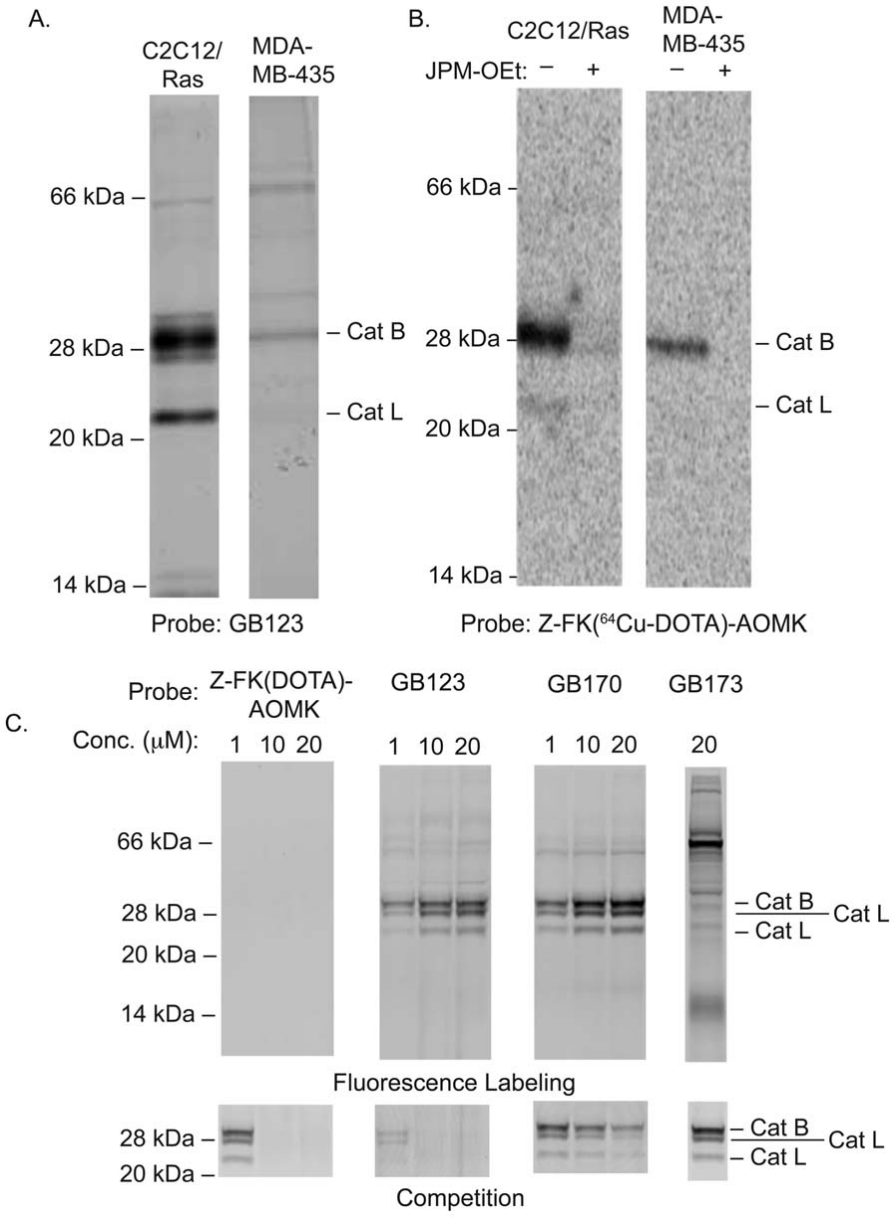
In vitro labeling properties of imaging probes. (**A**) Labeling of active cathepsins in the mouse myoblast cell line C2C12Ras, in the human breast cancer cell line MDA-MB-435, or in NIH3T3 mouse fibroblasts. Intact cells were labeled with GB123 for 1 hr followed by lysis and analysis of by SDS-PAGE and scanning of the gel for Cy5 fluorescence using a flatbed laser scanner (**B**) Labeling of cathepsins in C2C12/Ras and MDA-MB-435 cells. Cells were pretreated with 50 µM JPM-OEt (+) or with control DMSO (-) for 2 h and labeled by addition of ^64^Cu-Z-FK(DOTA)-AOMK (555 KBq, 15 µCi) for 2 h. Cells were collected, lysed and analyzed by SDS-PAGE. The labeled proteases were visulized by phosphorimaging with a Typhoon 9410 scanner. (**C**) NIH3T3 cells were incuabted with each probe at the indicated concentration for 1 hr followed by addition of a BODIPY-TMR-X-labeled GB111 probe. Directly labeling of target cathepsins by probes was obtained by SDS-PAGE followe by scanning of the gel using a flatbed laser scanner (Top gel). Inhibition of the same targets was observed by scanning of the gel for the BODIPY labeled probe using a Typhoon 9410 scanner (bottom gel).

We tested probes in the human breast cancer cell line MBA-MB-435 and a mouse myoblastoma cell line that had been transformed by over-expression of the ras oncogen (C2C12/Ras) [26]. Both of these cell lines were originally used for *in vivo* imaging studies with the NIRF-labeled cysteine cathepsin probes [21]. We labeled both cell lines with GB123 and found that cathepsin B and L activities of the C2C12/Ras cell line were higher than the activities observed for the same targets in the MDA-MB-435 cells (Figure 2A). Labeling of the same cells with ^64^Cu-Z-FK(DOTA)-AOMK confirmed that the DOTA probes produced a similar labeling pattern to that observed for GB123 (Figure 2B). In addition, the specificity of the Cu-labeled probe for the cysteine cathepsins was confirmed by the complete loss of labeling of the protease targets when cells were pretreated with the general cysteine cathepsin inhibitor JPM-OEt [25]. Finally, we compared the labeling properties of the dual fluorescent and PET tagged probe GB170 and GB173 to the original PET-only probe Z-FK(DOTA)-AOMK. Both GB170 and GB173 have fluorescent tags and can be used to directly label intact NIH 3T3 cells. For comparison with the non-fluorescently labeled probe, we labeled residual activity using a BODIPY-TMR labeled version of GB123 (Figure 2C). This analysis confirmed that the GB170 probe was able to effectively label active cathepsins B and L. Interestingly, the dual labeled probe GB170 showed slightly reduced potency in the competition assay compared to Z-FK(DOTA)-AOMK and GB123 even though it showed the most effective labeling of cathepsins as measured by direct labeling. GB173, which lacks the AOMK reactive group, failed to label active cathepsins and did not compete for labeling by the BODIPY-TMR GB123, making it an ideal negative control probe.

### Biodistribution of ^64^Cu-labeled probe in subcutaneous C2C12/Ras and MDA-MB-435 tumors

We next examined the overall biodistribution of the various ^64^Cu-labeled probes using athymic nude mice carrying subcutaneously grafted C2C12/Ras and MDA-MB-435 tumors. We measured uptake of Z-FK(DOTA)-AOMK in both the C2C12/Ras and MDA-MB-435 tumor models (Figure S2). This probe displayed rapid blood clearance, resulting in low blood and muscle uptake even at the early time points in both models. However, we did observe some accumulation of radioactivity in the C2C12/Ras tumors expressing high levels of cysteine cathepsins (0.35± 0.13%ID/g at 0.5 h p.i.). This activity remained in the tumors resulting in an uptake of 0.27 ±0.05%ID/g at 24 h p.i. For the MDA-MB-435 tumors with lower cysteine cathepsin activity, tumor uptake was significantly lower at each time point relative to the C2C12/Ras tumors (*P*<0.05). Furthermore, while moderate tumor-to-background ratios (tumor/blood 1.25 and tumor/muscle 5.64 at 2 h p.i.) were observed in the C2C12/Ras tumors, only low tumor-to-background ratios (tumor/blood 0.61 and tumor/muscle 3.03 at 2 h p.i.) were found for the MDA-MB-435 tumors. For both tumor models, ^64^Cu-Z-FK(DOTA)-AOMK displayed very low accumulation in most non-tumor tissues. The highest radioactivity was found in the liver, consistent with high endogenous expression of various cysteine cathepsins in this organ. Moderate renal accumulation was also observed at all times in both tumor models. These high levels of non-tumor signals may be partially due to loss of the copper radiotracer from the probe. This is an issue when using DOTA as a chelation agent. Subsequent studies will be aimed at using more optimal chelators and possibly other radio-tracers that can be secured to the probe through covalent modification. Regardless, the relatively low tumor accumulation of ^64^Cu-Z-FK(DOTA)-AOMK suggested that this probe is not optimal for *in vivo* applications.

For comparison, we measured the biodistribution of GB170 as well as its corresponding negative control GB173 in the C2C12/Ras tumor model (Figure S2). GB170 rapidly accumulated in the C2C12/Ras tumors (4.28± 0.91%ID/g), with higher levels of probe accumulation throughout all tissues compared to Z-FK(DOTA)-AOMK. The control probe GB173 showed some tumor accumulation (1.96± 0.73%ID/g) but this level was significantly (*P*<0.05) lower than the levels observed for GB170. Furthermore, we observed moderate to high tumor-to-background ratios (tumor/blood 3.1 and tumor/muscle 13.0 at 24 h p.i.) for GB170 in the C2C12/Ras tumors, while only low tumor-to-background ratios (tumor/blood 1.84 and tumor/muscle 4.4 at 24 h p.i.) for GB173.

### MicroPET imaging of cathepsin activity in tumor models

**Figure 3:**
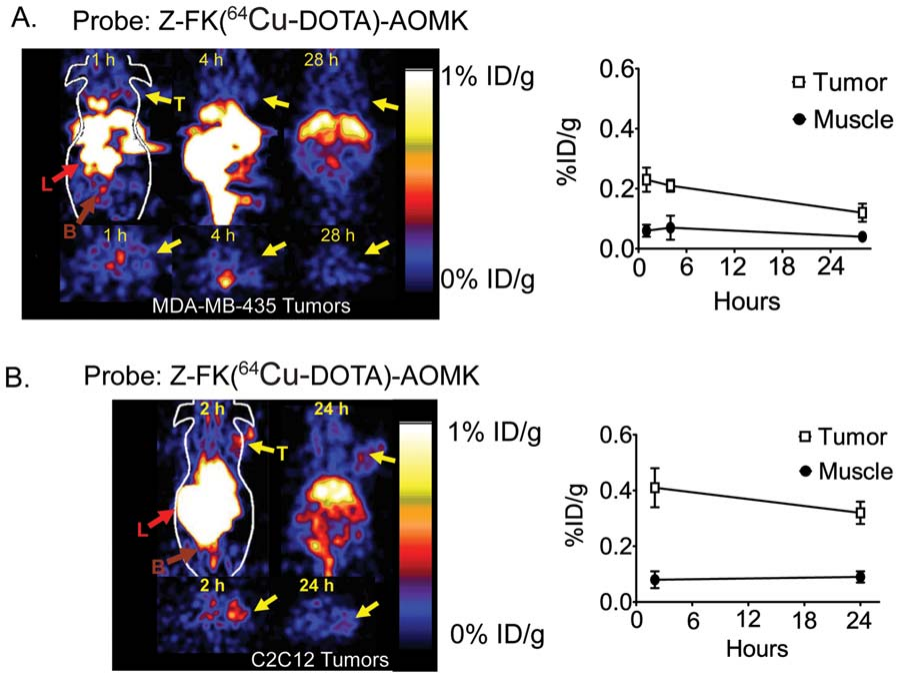
In vivo imaging of tumor-derived cathepsin activity using first generation activity-based probes. (**A**) Decay corrected coronal (top) and transaxial (bottom) microPET images of a nude mouse bearing MDA-MB-435 tumor at 1, 4 and 28 hours after tail vein injection of (5.55 MBq, 150 µCi) ^64^Cu-Z-FK(DOTA)-AOMK. The location of the tumor is indicated by arrows. Average values (n = 3) for tumor uptake (%ID/g±SD) relative to muscle signals at each time point are plotted (at right) (**B**) Decay corrected coronal (top) and transaxial (bottom) microPET images of a nude mouse bearing C2C12/Ras tumors at 2, and 24 hr after administration of (3.7 MBq, 100 µCi) ^64^Cu-Z-FK(DOTA)-AOMK. Arrows indicated location of tumors. Average values (n = 3) for tumor uptake (%ID/g±SD) relative to muscle signals at each time point are plotted (at right). For all images (T: tumor; L: liver; B: bladder).

We performed micro PET imaging of mice bearing the MDA-MB-435 and C2C12/Ras tumors using the ^64^Cu-Z-FK(DOTA)-AOMK probe (Figure 3). Although the probe signal in the liver and kidney is relatively high, the C2C12/Ras tumors could be clearly visualized with good tumor to contralateral background contrast of 2 at 24 h p.i. (Figure 3B). In contrast, we observed low tumor uptake and poor tumor to normal tissue contrast in the MDA-MB-435 tumors (Figure 3A). Quantification of the accumulation of the probe in the tumor and contralateral muscle tissue also showed that ^64^Cu-Z-FK(DOTA)-AOMK had higher tumor uptake and a higher tumor-to-muscle ratio in C2C12/Ras than in MDA-MB-435 (Figure 3B).

**Figure 4:**
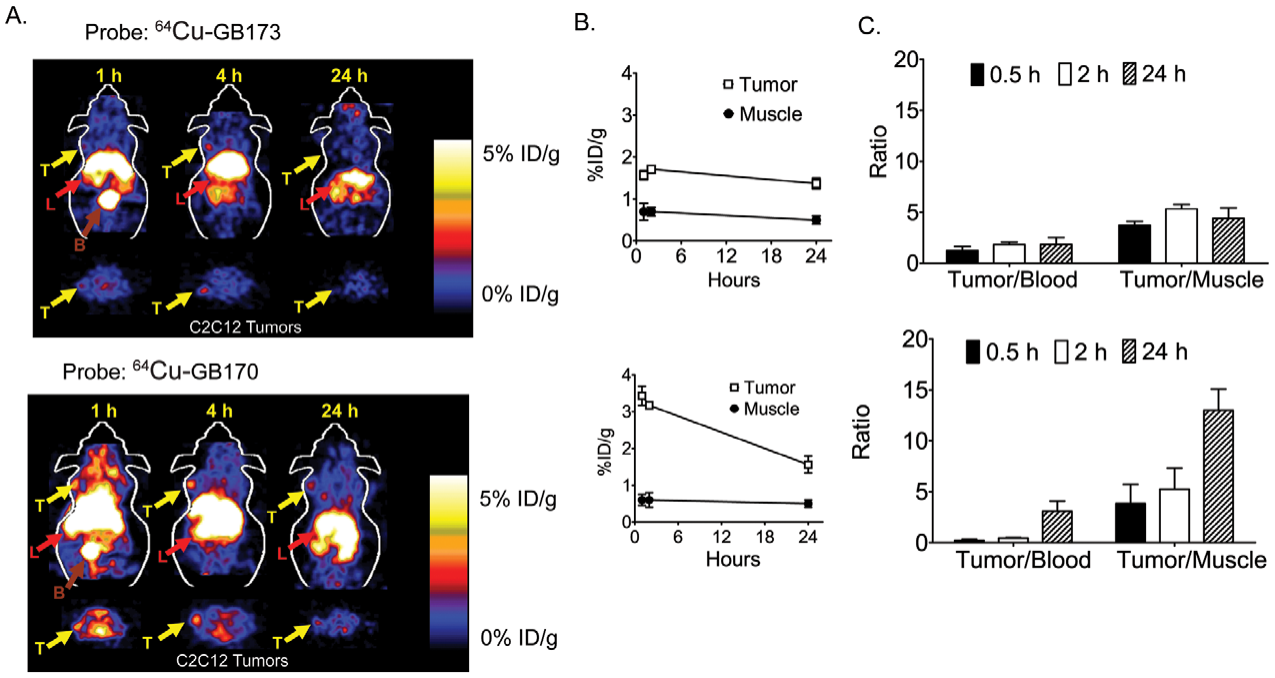
In vivo imaging of tumor-derived cathepsin activity using dual optical/PET imaging probes. (**A**) Coronal and transaxial decay corrected microPET images of an athymic nude mouse bearing C2C12/Ras tumor at different time points after tail vein injection of (1.85 MBq, 50 µCi) ^64^Cu-GB170 (bottom) or ^64^Cu-GB173 (top). The location of the tumor is indicated by arrows. (B) Tumor-to-normal tissues ratios of ^64^Cu-GB170 or ^64^Cu-GB173 in C2C12/Ras cancer bearing mice at different time points (n = 3 each). (C) Plots of average ratios (n = 3) of tumor to blood and tumor to muscle signals at the indicated time points. For all images (T: tumor; L: liver; B: bladder).

We next performed microPET imaging of mice bearing C2C12/Ras tumors using ^64^Cu-GB170 and the corresponding negative control, ^64^Cu-GB173 (Figure 4A). As expected, GB170 produced substantially higher signals that the ^64^Cu-Z-FK(DOTA)-AOMK probe with tumor to contralateral background contrast of 4 at 24 h p.i. In addition, ^64^Cu-GB173 showed only weak tumor uptake and poor tumor to normal tissue contrast. Specific probe uptake into tumors was confirmed by quantification of the tumor and muscle signals (Figure 4B) and by comparison of tumor to muscle and tumor to blood ratios (Figure 4C).

**Figure 5:**
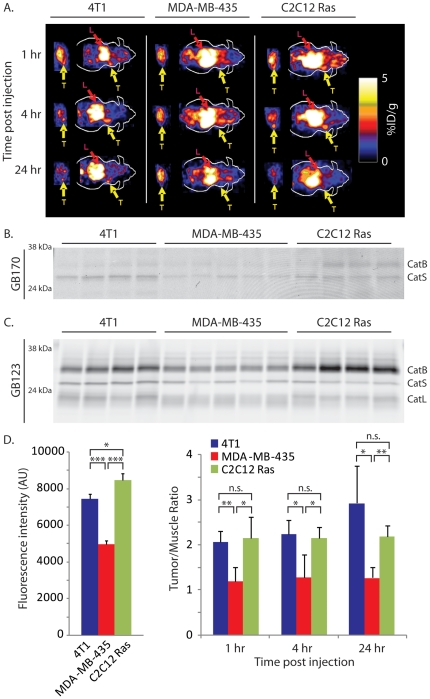
Probe signals in tumors correlate with levels of active cathepsins. (A) Representative coronal and transaxial decay corrected microPET images of athymic nude mice bearing 4T1, MDA-MB-435 and C2C12/Ras tumors at different time points after tail vein injection of (1.85 MBq, 50 µCi) ^64^Cu-GB170. Locations of tumors (T) and liver (L) are indicated. (B) SDS-PAGE and fluorescence scanning of tumor lysates from mice in (A) after the final 24 hr time point. The positions of fluorescently labeled cathepsins are indicated. (C) Determination of residual cathepsin activity in tumors from mice in (A) as measured by labeling of lysates with GB123 followed by SDS-PAGE and scanning of the gel with a flatbed laser scanner. (D) Comparison of the levels of probe levels in tumor relative to muscle as measured by PET (right bar graph) to the levels of active cathepsins as measured by quantification of gels in (C; left bar graph). The p-values are indicated and defined as follows: n.s. = not significant, *<0.05, **<0.01, ***<0.001.

While these data were suggestive that probe accumulation correlates with levels of active cathepsins in the tumor tissues, we needed to demonstrate this correlation by direct analysis of cathepsin actvity in samples after imaging. Therefore, we performed a study in which three different cell lines were used to generate xenograft tumors on nude mice. We chose the original MDA-MB-435 cells with low cathepsin activity and used the C2C12/Ras cells with high cathepsin levels. We also included a third tumor cell line (4T1) that also has high expression of cathepsins. We then performed PET imaging studies at various time points using the GB170 probe (Figure 5A). After quantification of tumor and muscle signals and subsequent biodistribution studies of collected tissues (Figure S3), we lysed the tumor tissues and analyzed both direct fluorescent labeling of cathepsins by the GB170 probe (Figure 5B) and residual cathepsin activities in the tumor lysates by addition of GB123 (Figure 5C). Subsequent quantification of the intensity of labeling of residual cathepsins by gel and comparison to levels of probe accumulation in tumors as measured by PET imaging, indicated a strong correlation between the two set of values (Figure 5D). Specifically, the two high expressors of cathepsins (4T1 and C2C12/Ras) showed indistinguishably (p = 0.7) high levels of cathepsin activity as measured by gel, while the tumors with low cathepsin activity (MDA-MB-435) showed significantly (p<0.05) reduced levels of cathepsins relative to either of the high cathepsin tumors. These levels matched the relative levels of probe observed in tumors by PET imaging, suggesting that probe signal is the result of retention at sites of high cysteine cathepsin activity.

**Figure 6:**
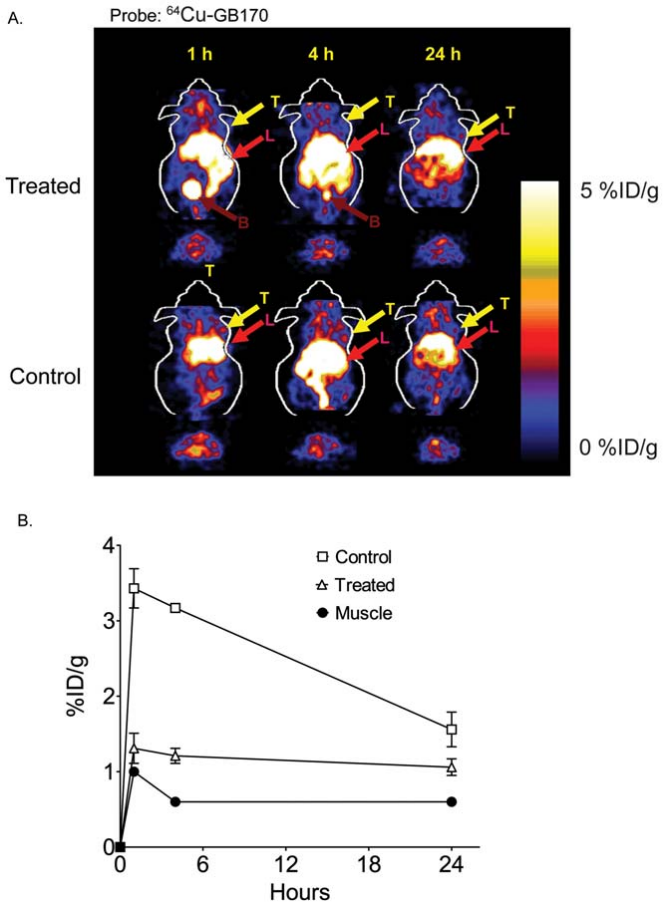
Inhibition of tumor-derived cathepsin activity can be visualized non-invasively using PET probes. Coronal and transaxial decay corrected microPET images of an athymic nude mouse bearing MDA-MB-231MFP tumors treated with the general cathepsin inhibitor K11777 (treated) or vehicle (control) followed by ^64^Cu-GB170 (1.85 MBq, 50 µCi). Images were taken at the indicated times after injection of the imaging probe. The location of the tumor is indicated by arrows. (B) Quantification of average levels (n = 3) of probe uptake in tumors at the various time points for control treated and inhibitor treated mice relative to uptake into muscle tissue. For all images (T: tumor; L: liver; B: bladder).

Finally, to confirm that the probe accumulation was due to modification of active cathepsins, we pre-treated C2C12/Ras tumor bearing mice with a broad-spectrum inhibitor of the cysteine cathepsins (Figure 6). This compound, K11777 was used in previous optical imaging studies to pre-block cathepsin activity [21]. MicroPET images show a marked drop in probe accumulation in tumors of mice treated with the inhibitor compared to those treated with the vehicle control (Figure 6A). Further quantification of tumor signals in vehicle and drug-treated animals compared to muscle signals confirmed the drop in specific signal as a result of inhibition of cysteine cathepsin activity (Figure 6B). Thus, virtually all of the probe signal observed above muscle background could be blocked by inhibition of the target proteases.

## Discussion

The cysteine cathepsins are potentially valuable molecular targets for tumor imaging because of their significant function in tumor progression, invasion and metastasis [1]-[7]. Although NIRF labeled probes that target this class of protease have been successfully applied to optical imaging applications [10], [13], [15], [16], [21], [22], radioactive probes have not been developed. Part of the reason for the lack of probes for radiological imaging applications is the difficulty in designing radioactive probes based on protease substrates. Therefore, in order to prepare new PET agents, we chose to make use of highly selective activity-based probes that covalently target proteases. The main disadvantage of using an inhibitor-based approach is the lack of amplification of signal that is obtained with substrate-based approaches. However, the high expression of cysteine cathepsins in tumor tissue [27], and the prior validation of these probes in optical imaging applications suggested that they would be potentially valuable for use in PET imaging.

We chose to use the validated peptide AOMK scaffold for development of our first generation PET probe. Simple conversion of the Z-FK-AOMK probe to the corresponding DOTA labeled analog Z-FK(DOTA)-AOMK (Figure 1B) allowed labeling with ^64^Cu. We selected ^64^Cu as a PET radiolabel because it can be readily produced using a medical cyclotron and the intermediate half-life of ^64^Cu makes it suitable for small molecule and peptide radiolabeling [28]-[30]. Overall labeling and biodistribution studies with ^64^Cu-Z-FK(DOTA)-AOMK indicated that the probe showed rapid clearance in blood but accumulated in tumor tissues with reasonable signal levels (Figure 3). Unfortunately, the probe showed overall low tumor uptake (only 0.35± 0.13%ID/g at 0.5 h p.i. in C2C12/Ras tumor model), possibly due to its lipophilicity (Figure S1) and loss of the fluorogenic Cy5 group found on the corresponding optical imaging probe. We have found that the addition of bulky aromatic groups on probes enhances their labeling of lysosomal proteases, possibly due to increased endocytosis. We therefore synthesized a PET probe in which the Cy5 fluorophore was retained on the P1 lysine sidechain and the DOTA tag for PET labeling was moved to the N-terminus of the peptide. Analysis of the GB170 probe in microPET imaging studies confirmed that the *in vivo* tumor uptake was clearly superior to ^64^Cu-Z-FK(DOTA)-AOMK in C2C12/Ras tumor bearing mice. We were also able to confirm that the signals observed for the GB170 probe were due to retention at the site of active cysteine cathepsins by showing correlation between imaging signals and protease activity as measured biochemically (Figure 5) as well as by showing a reduction in probe retention in mice pretreated with a broad-spectrum inhibitor of the cathepsins (Figure 6). These combined data confirm the selectivity of the tumor signals and also demonstrates the utility of PET probes for monitoring *in vivo* efficacy of small molecule drugs.

Although the probes reported here support the use of ABPs for PET imaging, significant work will be required to improve the probes. For example, release of free ^64^Cu from the radiolabeled compound or transchelation to other proteins may also be partially responsible for high background signal. Other chelators such as cross-bridged cyclam ligands may also be used in place of DOTA to improve the metal-chelate stability and subsequently improve the biodistribution of the probes [30], [31]. Regardless of the issues of probe background, the use of positron emitting labels allows application of tomographic methods to resolve signals in specific locations that are free from high protease background. In addition, unlike the NIRF versions of these ABPs the PET probes have the potential to be used to image tissues at much greater depths and with higher overall resolution [21]. The work presented here with ^64^Cu-labeled probes serves as the foundation for future studies using the more clinically suitable ^18^F tracer. We believe that cathepsin-targeted PET imaging agents should find broad applications once these agents become available to the nuclear medicine community.

## Materials and Methods

### Ethics Statement

All animal experiments were conducted according to relevant national and international guidelines and approved by the Stanford Institutional Animal Care and Use Committee (IACUC; approval number A3213-01). All experiments strictly followed the panel's specific guidelines regarding the care, treatment and euthanasia of animals used in the study.

### General

1,4,7,10-tetraazacyclododecane-1,4,7,10-tetraacetic acid (DOTA) was obtained from Macrocyclics Inc. (Richardson, TX). All other chemicals were obtained from Sigma-Aldrich Chemical Co. (St. Louis, MO). AOMK analogs, were prepared based on a reported synthetic method [20]. ^64^Cu was provided by the Department of Medical Physics, University of Wisconsin at Madison (Madison, WI). A CRC-15R PET dose calibrator (Capintec Inc., Ramsey, NJ) was used for all radioactivity measurements. Reverse phase high performance liquid chromatography (RP-HPLC) was performed on a Dionex Summit HPLC system (Dionex Corporation, Sunnyvale, CA) equipped with a 170U 4-Channel UV-Vis absorbance detector and radioactivity detector (Carroll & Ramsey Associates, model 105S, Berkeley, CA). UV detection wavelengths were 218 nm, 254 nm and 280 nm for all the experiments. Both semi-preparative (Vydac, Hesperia, CA. 218TP510-C18, 10 mm×250 mm) and analytical (Dionex, Sunnyvale, CA. Acclaim120 C18, 4.6 mm250 mm) RP-HPLC columns were used. The mobile phase was solvent A, 0.1% trifluoroacetic acid (TFA)/H_2_O, and solvent B, 0.1%TFA/acetonitrile. Matrix-assisted laser desorption/ionization time of flight mass spectrometry (MALDI-TOF-MS) were performed on a Perseptive Voyager-DE RP Biospectrometry instrument (Framingham, MA) by the Stanford Protein and Nucleic Acid Biotechnology Facility. Alpha cyano-4-hydroxy-cinnamic acid (α-CHCA, prepared as 10 g/L in 33.3% CH_3_CN : 33.3% EtOH : 33.3% H2O : 0.1% TFA) were used as the solid matrix. NIH3T3 mouse fibroblast cells was a generous gift from Dr. P. Jackson, Stanford University, CA. The tumorigenic murine skeletal myoblast cell line C2C12/Ras (retrovirally transduced with the ras oncogene) was a generous gift from Dr. Helen Blau, Stanford University, CA, human breast cancer MDA-MB-435 were a generous gift from Dr. X. Chen, Stanford University, CA. 4T1 cells were purchased ATCC. All the cells were original tested for microbial contamination, growth properties, morphology and species (COI assay) by the labs or vendor from where they were obtained and kept in culture for less than 6 months from resuscitation from frozen stocks. Female athymic nude mice (*nu/nu*) were purchased from Charles River Laboratories (Boston, MA).

### Chemistry and Radiochemistry

For details on the synthesis and radiolabeling of compounds please see Methods S1.

### Labeling Intact Tumor Cells with ^64^Cu Labeled ABP

C2C12/Ras and MDA-MB-435 cells (0.25 M each well) were seeded in a six-well plate one day before the study. Cells were pretreated with the general papain family protease inhibitor JPM-OEt (50 µM) [25] or with control DMSO (0.1%) for 1 h and labeled by addition of ^64^Cu-Z-FK(DOTA)-AOMK (555 KBq, 15 µCi) to culture medium for 2 h. Cells was then washed with PBS and lysed. Equal amounts of protein per lane were separated by 12% SDS-PAGE. The gel was dried and exposed to a phosphorimager screen. The labeled proteases were visualized by scanning of the phosphorimager screen with a Typhoon 9410 imager (GE Healthcare, Piscataway, NJ). The labeled bands were quantified with ImageJ 1.36b (public domain software from National Institute of Health).

### Labeling Intact Tumor Cells with AOMK analogs

NIH3T3 and C2C12 Ras cells were cultured in DMEM (GIBCO) supplemented with 10% fetal bovine serum (FBS; GIBCO), 100 units per mL penicillin and 100 µg per mL streptomycin (GIBCO). MDA-MB-435 cells were cultured in Leibovitz's L-15 medium (GIBCO) supplemented with 10%FBS, 100 units per mL penicillin and 100 µg per mL streptomycin (GIBCO). NIH3T3 cells (0.25 M each well) were seeded in a six-well plate one day before the study. Cells were pretreated with the general papain family protease inhibitor JPM-OEt (50 µM;[25] or with control DMSO (0.1%) for 1 h and labeled by addition of AOMK analogs to culture medium for 2 h. Cells was then washed with PBS and lysed. Equal amounts of protein per lane were separated by 12% SDS-PAGE. The gel was dried and exposed to a phosphorimager screen. The labeled proteases were visualized by scanning of the phosphorimager screen with a Typhoon 9410 imager (excitation/emission 633/680 nm) (GE Healthcare, Piscataway, NJ). For the Competition experiment, NIN3T3 cells were treated similarly as described above but were labeled with GB111-TMR-X (1 µM final concentration) after probe treatment and then lysed. Gells were scanned for fluorescence at 532/580 nm with a Typhoon scanner.

### Subcutaneous Tumor Model

All animal studies were carried out in compliance with Federal and local institutional rules for the conduct of animal experimentation. Female athymic nude mice (*nu/nu*) were obtained from Charles River Laboratories (Boston, MA) at 7–8 weeks old and kept under sterile conditions. The nude mice were inoculated subcutaneously in the right shoulder with 1×10^6^ cultured C2C12/Ras cells or 5×10^6^ MDA-MB-435 cells or 3×10^6^ cultured 4T1 cells. For inhibition studies, 3×10^6^ MDA-MB-231 MFP cells were used. When the tumors reached 0.5–0.8 cm in diameter, the tumor bearing mice were were used for *in vivo* PET imaging studies (see below).

### MicroPET Imaging

PET imaging of tumor-bearing mice will be performed on a microPET R4 rodent model scanner (Siemens Medical Solutions USA, Inc., Knoxville, TN). The mice bearing C2C12/Ras and MDA-MB-435 tumors were injected with 740 KBq (20 µCi) ^64^Cu AOMK probe via the tail vein. At different times after injection, 5 or 10-min static scans were obtained and the images were reconstructed by a two-dimensional ordered subsets expectation maximum (OSEM) algorithm. Regions of interest (ROIs) were then drawn over the tumor on decay-corrected whole-body coronal images. The mean counts per pixel per minute were obtained from the ROI and converted to counts per milliliter per minute by using a calibration constant. By assuming a tissue density of 1 g/mL, the ROIs were converted to counts/g/min. An image ROI-derived %ID/g of tissue was then determined by dividing counts per gram per minute with injected dose (ID). No attenuation correction was performed.

### Biodistribution Studies

Mice bearing xenografts were injected with 740 KBq (20 µCi) of ^64^Cu labeled tracer through the tail vein and sacrificed at different time points after injection. Tumor and normal tissues were removed and weighed, and radioactivity was measured by gamma-counter. The radioactivity uptake in the tumor and normal tissues was expressed as a percentage of the injected radioactive dose per gram of tissue (%ID/g). To inhibit the *in vivo* cathepsin activity, K11777 was injected intraperitoneally at the dose of 100 mg/kg/day in 40% DMSO/sterile PBS in a final volume of 100 µL for 5 days before the imaging sessions [21].

### Biochemical analysis of cathepsin activity in tumors

Tumors were removed after the 24 hr time point and homogenized in citrate buffer (50mM Citrate pH 5.5, 5 mM DDT, 0.5% CHAPS, 0.1% Triton X). Total protein (40 µg) was labeled with GB123 (1 µM final concentration) for 1 hr at 37°C, the proteins were resolved on SDS-PAGE (15%). Labeled proteases were visualized by scanning the gel with a Typhoon 9410 imager (excitation/emission 633/680 nm) (GE Healthcare, Piscataway, NJ). Labeling intensities were quantified using Image J software.

### Statistical Method

Statistical analysis was performed using the Student's *t*-test for unpaired data. A 95% confidence level was chosen to determine the significance between groups, with *P*<0.05 being significantly different.

## Supporting Information

Figure S1 **Characterization and purification PET probes.** HPLC radiochromatogram of purified (A) ^64^Cu-Z-FK(DOTA)-AOMK (B) ^64^Cu-GB170, and (C) ^64^Cu-GB173.
(DOC)

Figure S2 **Biodistribution of probes** ***in vivo*** **.** Biodistribution results of ^64^Cu-Z-FK(DOTA)-AOMK in (**A**) C2C12/Ras (top) or MDA-MB-435 (bottom) cancer bearing mouse models as well for (**B**) ^64^Cu-GB170 (top) and ^64^Cu-GB173 (bottom) in C2C12/ras tumor bearing mice. Data are expressed as the percentage administered activity (injected dose) per gram of tissue (%ID/g) after intravenous injection of 740 kBq (20 µCi) of^ 64^Cu-Z-FK(DOTA)-AOMK at 0.5, 2, and 24 h pi (n = 3). Significant lower tumor uptake and tumor/blood, tumor/muscle ratio in MDA-MB-435 breast cancer (*P*<0.05) were observed.
(DOC)

Figure S3 **Biodistribution of ^64^Cu-GB170 in tumor bearing mice.** N = 4 for each.
(DOC)

Methods S1 **Chemistry and Radiochemistry.**
(DOC)

## References

[1] Cavallo-Medved D, Sloane BF (2003). Cell-surface cathepsin B: understanding its functional significance.. Curr Top Dev Biol.

[2] Jedeszko C, Sloane BF (2004). Cysteine cathepsins in human cancer.. Biol Chem.

[3] Koblinski JE, Ahram M, Sloane BF (2000). Unraveling the role of proteases in cancer.. Clin Chim Acta.

[4] Mohamed MM, Sloane BF (2006). Cysteine cathepsins: multifunctional enzymes in cancer.. Nat Rev Cancer.

[5] Sloane BF, Sameni M, Podgorski I, Cavallo-Medved D, Moin K (2006). Functional imaging of tumor proteolysis.. Annu Rev Pharmacol Toxicol.

[6] Sloane BF, Yan S, Podgorski I, Linebaugh BE, Cher ML (2005). Cathepsin B and tumor proteolysis: contribution of the tumor microenvironment.. Semin Cancer Biol.

[7] Joyce JA, Hanahan D (2004). Multiple roles for cysteine cathepsins in cancer.. Cell Cycle.

[8] Blum G, Mullins SR, Keren K, Fonovic M, Jedeszko C (2005). Dynamic imaging of protease activity with fluorescently quenched activity-based probes.. Nat Chem Biol.

[9] Campo E, Munoz J, Miquel R, Palacin A, Cardesa A (1994). Cathepsin B expression in colorectal carcinomas correlates with tumor progression and shortened patient survival.. Am J Pathol.

[10] Figueiredo JL, Alencar H, Weissleder R, Mahmood U (2006). Near infrared thoracoscopy of tumoral protease activity for improved detection of peripheral lung cancer.. Int J Cancer.

[11] Gambhir SS (2002). Molecular imaging of cancer with positron emission tomography.. Nat Rev Cancer.

[12] Berger AB, Vitorino PM, Bogyo M (2004). Activity-based protein profiling: applications to biomarker discovery, in vivo imaging and drug discovery.. Am J Pharmacogenomics.

[13] Bogdanov AA, Lin CP, Simonova M, Matuszewski L, Weissleder R (2002). Cellular activation of the self-quenched fluorescent reporter probe in tumor microenvironment.. Neoplasia.

[14] Baruch A, Jeffery DA, Bogyo M (2004). Enzyme activity–it's all about image.. Trends Cell Biol.

[15] Bremer C, Tung CH, Bogdanov A, Weissleder R (2002). Imaging of differential protease expression in breast cancers for detection of aggressive tumor phenotypes.. Radiology.

[16] Mahmood U, Tung CH, Bogdanov A, Weissleder R (1999). Near-infrared optical imaging of protease activity for tumor detection.. Radiology.

[17] Jiang T, Olson ES, Nguyen QT, Roy M, Jennings PA (2004). Tumor imaging by means of proteolytic activation of cell-penetrating peptides.. Proc Natl Acad Sci U S A.

[18] Olson ES, Aguilera TA, Jiang T, Ellies LG, Nguyen QT (2009). In vivo characterization of activatable cell penetrating peptides for targeting protease activity in cancer.. Integr Biol (Camb).

[19] Olson ES, Jiang T, Aguilera TA, Nguyen QT, Ellies LG (2010). Activatable cell penetrating peptides linked to nanoparticles as dual probes for in vivo fluorescence and MR imaging of proteases.. Proc Natl Acad Sci U S A.

[20] Kato D, Boatright KM, Berger AB, Nazif T, Blum G (2005). Activity-based probes that target diverse cysteine protease families.. Nat Chem Biol.

[21] Blum G, von Degenfeld G, Merchant MJ, Blau HM, Bogyo M (2007). Noninvasive optical imaging of cysteine protease activity using fluorescently quenched activity-based probes.. Nat Chem Biol.

[22] Edgington LE, Berger AB, Blum G, Albrow VE, Paulick MG (2009). Noninvasive optical imaging of apoptosis by caspase-targeted activity-based probes.. Nat Med.

[23] Lee M, Fridman R, Mobashery S (2004). Extracellular proteases as targets for treatment of cancer metastases.. Chem Soc Rev.

[24] Lim IT, Meroueh SO, Lee M, Heeg MJ, Mobashery S (2004). Strategy in inhibition of cathepsin B, a target in tumor invasion and metastasis.. J Am Chem Soc.

[25] Joyce JA, Baruch A, Chehade K, Meyer-Morse N, Giraudo E (2004). Cathepsin cysteine proteases are effectors of invasive growth and angiogenesis during multistage tumorigenesis.. Cancer Cell.

[26] D'Orazi G, Marchetti A, Crescenzi M, Coen S, Sacchi A (2000). Exogenous wt-p53 protein is active in transformed cells but not in their non-transformed counterparts: implications for cancer gene therapy without tumor targeting.. J Gene Med.

[27] Eijan AM, Sandes EO, Riveros MD, Thompson S, Pasik L (2003). High expression of cathepsin B in transitional bladder carcinoma correlates with tumor invasion.. Cancer.

[28] Cheng Z, Xiong Z, Subbarayan M, Chen X, Gambhir SS (2007). 64Cu-labeled alpha-melanocyte-stimulating hormone analog for microPET imaging of melanocortin 1 receptor expression.. Bioconjug Chem.

[29] Anderson CJ, Dehdashti F, Cutler PD, Schwarz SW, Laforest R (2001). 64Cu-TETA-octreotide as a PET imaging agent for patients with neuroendocrine tumors.. J Nucl Med.

[30] Sun X, Kim J, Martell AE, Welch MJ, Anderson CJ (2004). In vivo evaluation of copper-64-labeled monooxo-tetraazamacrocyclic ligands.. Nucl Med Biol.

[31] Boswell CA, Sun X, Niu W, Weisman GR, Wong EH (2004). Comparative in vivo stability of copper-64-labeled cross-bridged and conventional tetraazamacrocyclic complexes.. J Med Chem.

