# Supplementary material for: Non-Invasive Imaging of Cysteine Cathepsin Activity in Solid Tumors Using a 64Cu-Labeled Activity-Based Probe
Source: PLoS One. 2011 Nov 21;6(11):e28029. doi: 10.1371/journal.pone.0028029 (PMC3221694; doi:10.1371/journal.pone.0028029)
Supplement: Figure S1 — Characterization and purification PET probes. HPLC radiochromatogram of purified (A) 64Cu-Z-FK(DOTA)-AOMK (B) 64Cu-GB170, and (C) 64Cu-GB173. (DOC) [file pone.0028029.s001.doc]

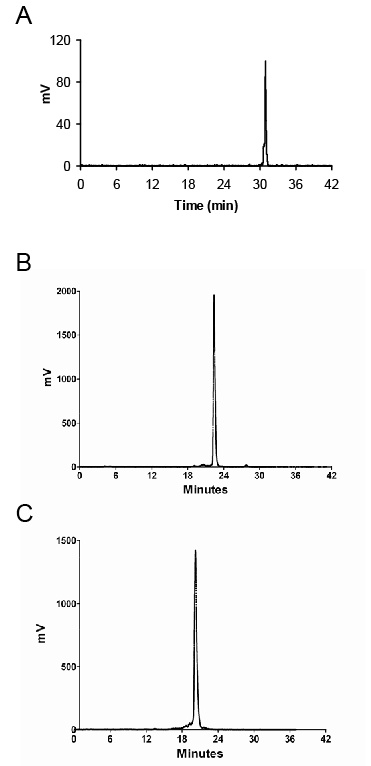


**Figure S1.** Characterization and purification PET probes. HPLC radiochromatogram of purified (A) ^64^Cu-Z-FK(DOTA)-AOMK (B) ^64^Cu-GB170, and (C) ^64^Cu-GB173.
