# Supplementary material for: Non-Invasive Imaging of Cysteine Cathepsin Activity in Solid Tumors Using a 64Cu-Labeled Activity-Based Probe
Source: PLoS One. 2011 Nov 21;6(11):e28029. doi: 10.1371/journal.pone.0028029 (PMC3221694; doi:10.1371/journal.pone.0028029)
Supplement: Figure S2 — Biodistribution of probes in vivo . Biodistribution results of 64Cu-Z-FK(DOTA)-AOMK in (A) C2C12/Ras (top) or MDA-MB-435 (bottom) cancer bearing mouse models as well for (B) 64Cu-GB170 (top) and 64Cu-GB173 (bottom) in C2C12/ras tumor bearing mice. Data are expressed as the percentage administered activity (injected dose) per gram of tissue (%ID/g) after intravenous injection of 740 kBq (20 µCi) of 64Cu-Z-FK(DOTA)-AOMK at 0.5, 2, and 24 h pi (n = 3). Significant lower tumor uptake and tumor/blood, tumor/muscle ratio in MDA-MB-435 breast cancer (P<0.05) were observed. (DOC) [file pone.0028029.s002.doc]

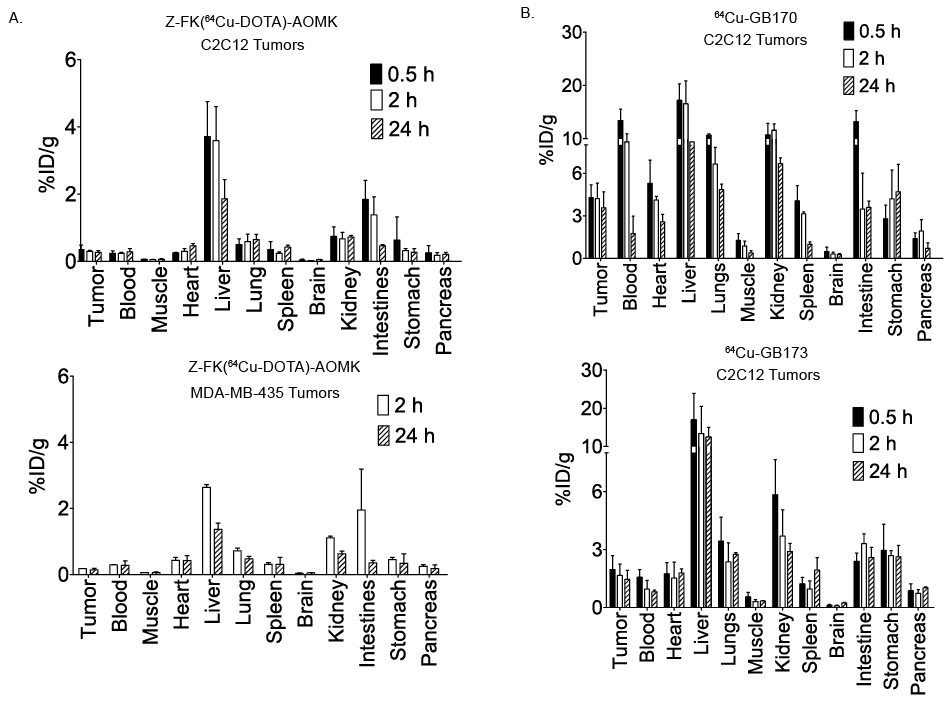


**Figure S2.** Biodistribution of probes *in vivo*. Biodistribution results of ^64^Cu-Z-FK(DOTA)-AOMK in (**A**) C2C12/Ras (top) or MDA-MB-435 (bottom) cancer bearing mouse models as well for (**B**) ^64^Cu-GB170 (top) and ^64^Cu-GB173 (bottom) in C2C12/ras tumor bearing mice. Data are expressed as the percentage administered activity (injected dose) per gram of tissue (%ID/g) after intravenous injection of 740 kBq (20 μCi) of ^64^Cu-Z-FK(DOTA)-AOMK at 0.5, 2, and 24 h pi (n = 3). Significant lower tumor uptake and tumor/blood, tumor/muscle ratio in MDA-MB-435 breast cancer (*P* < 0.05) were observed.
