# Supplementary material for: Non-Invasive Imaging of Cysteine Cathepsin Activity in Solid Tumors Using a 64Cu-Labeled Activity-Based Probe
Source: PLoS One. 2011 Nov 21;6(11):e28029. doi: 10.1371/journal.pone.0028029 (PMC3221694; doi:10.1371/journal.pone.0028029)
Supplement: Figure S3 — Biodistribution of 64Cu-GB170 in tumor bearing mice. N = 4 for each. (DOC) [file pone.0028029.s003.doc]

C2C12/Ras Tumor bearing mice MDA-MB-435 Tumor Bearing mice

| Organ | Mean %ID/gm | STD Dev |
| --- | --- | --- |
| Kidneys | 2.04 | 0.13 |
| Blood | 0.37 | 0.04 |
| Heart | 1.00 | 0.05 |
| Liver | 1.19 | 0.16 |
| Lungs | 3.02 | 0.09 |
| Spleen | 0.84 | 0.32 |
| Pancreas | 0.56 | 0.06 |
| Stomach | 1.34 | 0.37 |
| Brain | 0.10 | 0.01 |
| Intestine | 1.27 | 0.16 |
| Muscle | 0.18 | 0.04 |
| Bone | 0.39 | 0.02 |
| **Tumor** | **0.74** | **0.06** |

| Organ | Mean %ID/gm | STD Dev |
| --- | --- | --- |
| Kidneys | 2.10 | 0.16 |
| Blood | 0.49 | 0.27 |
| Heart | 1.08 | 0.26 |
| Liver | 1.36 | 0.16 |
| Lungs | 3.62 | 0.40 |
| Spleen | 1.11 | 0.65 |
| Pancreas | 0.53 | 0.09 |
| Stomach | 1.08 | 0.06 |
| Brain | 0.10 | 0.02 |
| Intestine | 1.28 | 0.27 |
| Muscle | 0.31 | 0.24 |
| Bone | 0.37 | 0.16 |
| **Tumor** | **0.55** | **0.08** |

4T1 Tumor bearing mice

| Organ | Mean %ID/gm | STD Dev |
| --- | --- | --- |
| Kidneys | 2.08 | 0.26 |
| Blood | 0.39 | 0.10 |
| Heart | 0.95 | 0.12 |
| Liver | 1.40 | 0.22 |
| Lungs | 3.98 | 0.54 |
| Spleen | 0.89 | 0.11 |
| Pancreas | 0.56 | 0.16 |
| Stomach | 1.25 | 0.20 |
| Brain | 0.10 | 0.02 |
| Intestine | 1.36 | 0.23 |
| Muscle | 0.17 | 0.01 |
| Bone | 0.29 | 0.05 |
| **Tumor** | **0.72** | **0.20** |

**Figure S3** – Biodistribution of ^64^Cu-GB170 in tumor bearing mice. N=4 for each
