# Supplementary material for: Non-Invasive Imaging of Cysteine Cathepsin Activity in Solid Tumors Using a 64Cu-Labeled Activity-Based Probe
Source: PLoS One. 2011 Nov 21;6(11):e28029. doi: 10.1371/journal.pone.0028029 (PMC3221694; doi:10.1371/journal.pone.0028029)
Supplement: Methods S1 — Chemistry and Radiochemistry. (DOC) [file pone.0028029.s004.doc]

**Methods S1**

**Chemistry and Radiochemistry**

DOTA was conjugated to the Z-FK-AOMK intermediate in a manner similar to that described previously (1). Briefly, DOTA, 1-ethyl-3-[3-(dimethylamino)propyl]carbodiimide (EDC), and *N-*hydroxysulfonosuccinimide (SNHS) at a molar ratio of 1:1:0.8 were mixed and incubated at 4°C for 30 min (pH = 5.5). Free amine containing AOMK analogs were then added to the *in situ* prepared sulfosuccinimidyl ester of DOTA (DOTA-OSSu) in a theoretic stoichiometry of 5:1. The reaction solution was mixed and reacted at 4°C overnight (pH 8.5–9.0). The resulting conjugate was then purified by HPLC on a semipreparative C-18 column. The flow rate was 3 mL/min, with the mobile phase starting from 95% solvent A and 5% solvent B (0–3 min) to 35% solvent A and 65% solvent B at 33 min, then going to 85% solvent B and 15% solvent A (33–36 min), maintaining this solvent composition for another 3 min (36–39 min) and returning to initial solvent composition by 42 min. Fractions were collected and lyophilized. The target product was characterized by MALDI-TOF-MS. The measured molecular weight (MW) was consistent with the expected MW: *m/z* = 960.5 for [M + H]^+^ (C_49_H_66_N_7_O_13_, Calculated MW = 960.5). For the GB170, the peptide AOMK was synthesized using solid phase peptide synthesis as reported (1) and the DOTA-tris t-butyl ester was coupled to the N-terminal amine on resin. The t-butyl ester protecting groups on the DOTA were removed during cleavage of the peptide from the resin. The resulting DOTA-labeled peptides were purified by HPLC and the Cy5 fluorophore was coupled using the reported methods (1). The measured molecular weight (MW) was consistent with the expected MW: *m/z* = 1612.8 for [M + H]^+^ (C_83_H_107_N_10_O_19_S_2_, Calculated MW = 1612.7). For the control GB173, the peptide amide was synthesized using standard solid phase peptide sysnthesis on Rink Amide resin. The measured molecular weight (MW) was consistent with the expected MW: *m/z* = 1465.1 for [M + H]^+^ (C_73_H_98_N_11_O_17_S_2_, Calculated MW = 1465.6).

The AOMK analogs were radiolabeled with ^64^Cu by addition of 185 MBq (5 mCi) ^64^CuCl_2_ (2 μg compound per 37 MBq ^64^Cu) in 0.1 N NaOAc (pH 5.5) buffer followed by a 1 hour incubation at 50°C. The radiolabeled complex was then purified by analytical radio-HPLC. The reaction solution was injected into an analytical radio-HPLC using the same elution gradient and flow rate as the one used for the cold ZFK(DOTA)-AOMK or GB170 and GB173 analysis. Purified radiolabeled peptide was dried by rotary evaporation. The radiolabeled compound was reconstituted in phosphate-buffered saline (PBS) and passed through a 0.22 μm Millipore filter into a sterile vial for *in vitro* and animal experiments.

**References**

1. Cheng Z, Xiong Z, Subbarayan M, Chen X, Gambhir SS. 64Cu-labeled alpha-melanocyte-stimulating hormone analog for microPET imaging of melanocortin 1 receptor expression. Bioconjug Chem 2007;18(3):765-72.
